# Sterile and Dual-Porous Aerogels Scaffolds Obtained through a Multistep Supercritical CO_2_-Based Approach

**DOI:** 10.3390/molecules24050871

**Published:** 2019-03-01

**Authors:** Víctor Santos-Rosales, Inés Ardao, Carmen Alvarez-Lorenzo, Nilza Ribeiro, Ana L. Oliveira, Carlos A. García-González

**Affiliations:** 1 Departamento de Farmacología, Farmacia y Tecnología Farmacéutica, R+D Pharma group (GI-1645), Facultad de Farmacia and Health Research Institute of Santiago de Compostela (IDIS), Universidade de Santiago de Compostela, E-15782 Santiago de Compostela, Spain; victor.santos.rosales@rai.usc.es (V.S.-R.); carmen.alvarez.lorenzo@usc.es (C.A.-L.); 2 BioFarma Research group, Centro Singular de Investigación en Medicina Molecular y Enfermedades Crónicas (CiMUS), Universidade de Santiago de Compostela, E-15782 Santiago de Compostela, Spain; ines.ardao@usc.es; 3 CBQF-Centro de Biotecnologia e Química Fina-Laboratório Associado, Escola Superior de Biotecnologia, Universidade Católica Portuguesa, 4200-375 Porto, Portugal; nribeiro@porto.ucp.pt (N.R.); aloliveira@porto.ucp.pt (A.L.O.)

**Keywords:** starch aerogels, zein, supercritical sterilization, supercritical CO_2_, regenerative medicine

## Abstract

Aerogels from natural polymers are endowed with attractive textural and biological properties for biomedical applications due to their high open mesoporosity, low density, and reduced toxicity. Nevertheless, the lack of macroporosity in the aerogel structure and of a sterilization method suitable for these materials restrict their use for regenerative medicine purposes and prompt the research on getting ready-to-implant dual (macro + meso)porous aerogels. In this work, zein, a family of proteins present in materials for tissue engineering, was evaluated as a sacrificial porogen to obtain macroporous starch aerogels. This approach was particularly advantageous since it could be integrated in the conventional aerogel processing method without extra leaching steps. Physicochemical, morphological, and mechanical characterization were performed to study the effect of porogen zein at various proportions (0:1, 1:2, and 1:1 zein:starch weight ratio) on the properties of the obtained starch-based aerogels. From a forward-looking perspective for its clinical application, a supercritical CO_2_ sterilization treatment was implemented for these aerogels. The sterilization efficacy and the influence of the treatment on the aerogel final properties were evaluated mainly in terms of absence of microbial growth, cytocompatibility, as well as physicochemical, structural, and mechanical modifications.

## 1. Introduction

The increase in life expectancy, the popularization of physical activity in society, and the high obesity ratios have strongly increased the incidence of bone diseases and traumatic fractures. The scarcity of donor grafts and the associated clinical complications (autoimmune response) even with autologous procedures (risk of surgeries sequalae and donor morbility), have prompted the development of innovative synthetic grafts (scaffolds) [1]. A 3D hierarchical and interconnected porous structure with surface roughness are among the desired features for the scaffolds to favor the vascularization and growth of the damaged tissue [1,2]. Moreover, scaffolds should temporarily surrogate natural tissue, so they should present an appropriate mechanical behavior according to the anatomical target [3,4]. Finally, the employed materials must ensure biocompatibility and suitable biodegradation rates to avoid immunogenic responses and malformations, respectively.

Aerogels are lightweight materials with outstanding textural properties that allow the manufacturing of multishape structures from different inorganic and organic sources [5,6]. Since their invention back in the 1930s [7], many different fields (e.g., electrical and chemical applications, aerospace, and building industries) [8,9,10,11] have taken advantage of their low acoustic and thermal conductivity, extremely low density, and high open porosity. Namely, the “bio-based aerogels” term was coined in the 21st century [12] to denote polysaccharide and protein-based aerogels from different sources and with promising biomedical applications [6,13]. Current research on bio-based aerogels has been mainly focused on drug delivery systems [6] with only few examples in the spotlight of the regenerative medicine [4,14,15,16]. For the latter case, a fast ingress of body fluids in the aerogel scaffold will take place by capillarity just after implantation due their initial dry state thus accelerating integration of the material. Starch aerogels are of particular interest for regenerative medicine due to their low toxicity, thermal stability, and abundance [6,17]. There are several FDA-compliant starch varieties that have demonstrated the capacity of promotion of cell adhesion and growth and of the phenotypic expression of osteoblastic markers [18].

Aerogels display a defined microstructure (micro, meso, or mixed porosity) [19] mimicking the native extracellular matrix [4], but they commonly lack of pores in the macroscale, which are required to favor cell penetration and colonization as well as the supply of nutrients towards the cells and the removal of waste metabolic products [20]. Macropores in aerogels can be induced by the addition of porogens as sacrificial particles (e.g., salts, sugar, or paraffin spheres) of defined sizes, typically by mixing with the polymeric solution before the gelation step [21,22,23,24]. Nevertheless, this practice involves an extra leaching step to remove the porogen from the gel structure, thus increasing the process complexity and the overall processing times as well as reducing the process yield. Hence, the identification of alternative porogens able to be integrated in the “classical processing approach of aerogels” is of interest for the sake of economy of the process.

Zeins are the main storage proteins found in the seeds of corn, and are widely used in a variety of products [25]. Specifically, zein could be used in tissue engineering due to its proven biocompatibility [26] along with its biological effect in the in vivo promotion of mesenchymal stem cell adhesion and proliferation in scaffolds, leading to increased vascularization and osteogenesis [27,28]. The insolubility in water is one of the most defining characteristic of zein, and high temperatures and different ethanol concentrations are typically employed for its extraction from the corn seeds [25]. The biological properties of zein and its reverse solubility behavior in water/ethanol with respect to starch [25,29] renders it an attractive porogen for the manufacturing of macroporous starch aerogels. Unlike other porogens reported in the literature, the removal of zein porogen from the starch gels can be integrated in the classical aerogel processing since the typical solvent exchange to ethanol for the hydrogel-alcogel transition can be also exploited for zein leaching. Overall, the use of zein as a porogen opens new ways to obtain aerogels endowed with macroporosity and compatible with biomedical purposes while avoiding extra processing leaching steps.

Sterilization of aerogels is a remaining remarkable challenge. Current sterilization techniques for medical devices (heat, irradiation, and chemical treatment) may not be suitable for aerogels since they do not have enough penetration capacity to reach the inner part of porous structures or can alter the physicochemical properties of the biomaterial [30]. It has been shown that supercritical (sc)CO_2_ can inactivate a wide range of microorganisms, including bacterial spores [31]. Temperature and pressure are considered the most important factors affecting the microorganism viability under scCO_2_. Therefore, the use of scCO_2_ also as a sterilization method for aerogels emerges as an attractive and still unexplored alternative due to the mild operating conditions and the lack or low content of additives required [30,32]. From a processing point of view, the combination of the manufacturing and sterilization of tailored polysaccharide aerogels for regenerative medicine under a same supercritical fluid-based technological platform would reduce the overall processing times, and consequently, the cost.

In this work, supercritical fluid technology was challenged to obtain sterile starch aerogels endowed with a combined macro and mesoporosity. Firstly, zein particles were evaluated as a biocompatible porogen to provide macroporosity to corn starch-based aerogels for further use in biomedical applications. The role of this porogen under different contents on the resulting aerogels was assessed regarding their morphological, textural, and mechanical properties. Then, a scCO_2_ sterilization method was implemented and evaluated for the treatment of aerogels for the first time. The sterilization efficacies of the treatment, its effect on the textural and mechanical properties of the aerogels, as well as the cytocompatibility of the sterile materials with bone marrow-derived mesenchymal stem cells (MSCs) were studied.

## 2. Results and Discussion

### 2.1. Starch-Based Aerogels Preparation

Pure corn starch aerogels were prepared following a conventional aerogel processing [33]: (1) Hydrogel formation, (2) a dehydration stage where water is substituted for ethanol, and (3) the subsequent supercritical drying. Cylindrical, lightweight corn starch aerogels were thus obtained ([Z0] in Table 1). A 10% (*w*/*w*) corn starch ratio was chosen as a trade-off solution between suitable aerogel mechanical properties (below 7% (*w*/*w*) resulted in fragile gels) and sol-state fluidity to dose the cylindrical molds with reproducibility and to get homogeneous structures (over 15% (*w*/*w*) turn highly viscous dispersions) [17].

**Figure 1:**
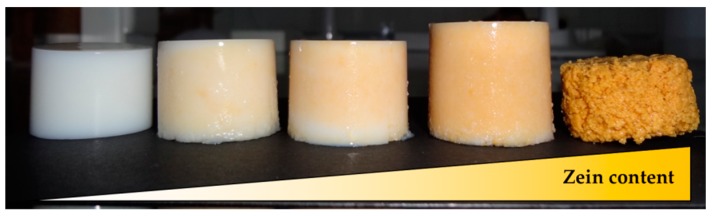
Appearance of starch-zein gels obtained from aqueous solutions with 10 wt.% starch and increasing zein content (from left to right, 0:1, 1:5, 1:3, 1:1, and 1:0 zein-to-starch weight ratio).

Using the same processing approach as with starch aerogels [Z0], cylindrical, white, and monolithic starch aerogels with dual porosity were obtained through the addition of zein as a sacrificial porogen and regardless of the zein content used ([Z1] and [Z2] in Table 1). After gelation, starch gels had the zein homogeneously distributed throughout the entire monolithic structure without visually detecting the presence of lumps. An intensification of the yellow coloration in the starch-zein gels, characteristic of the xanthophyllic pigments from zein, was observed as the zein content increased (Figure 1). Stable gels were not obtained from pure zein dispersions (Figure 1, right). 

The dehydration stage (solvent exchange) and the porogen leaching stage were brought together taking advantage of the differing solubility behavior in water and in ethanol of zein (insoluble in water) and corn starch (insoluble in ethanol) [25,34]. Previous attempts in incorporating porogens in aerogels [21,22,23,24] resulted in additional processing steps involving the use of water [21,22,23] or organic solvents like hexane [24] that should be specifically selected to leach the porogen from the material before the solvent exchange step to ethanol.

Corn starch aqueous solutions of 10% (*w*/*w*) containing zein in 0:1 ([Z0] aerogel), 1:2 ([Z1]), and 1:1 ([Z2]) weight ratios with respect to the starch content were chosen as optimal for evaluating the porogen addition effect, regarding suitable gel integrity, and the required midrange times for the porogen leaching. All the tested starch aerogel formulations had a volume reduction (shrinkage) after the serial water-to-ethanol solvent exchanges and the supercritical drying steps with respect to the initial volume of the gel dispersions (ΔV in Table 1). The volume reduction observed for the starch aerogel formulation [Z0] (39.1% in Table 1) was a similar value to those reported by other authors [33,35,36]. The gel shrinkage was reported to mainly occur during the solvent exchange step and to decrease with the starch content of the formulation [33]. The porogen addition displayed a remarkable decreasing effect on the shrinkage values with a two-fold reduction for the aerogel with higher zein content ([Z2] in Table 1). The presence of hydrophobic zein in the gel matrix during the aerogel processing might provide a certain improvement in the mechanical stability of the gel resulting in reduced overall aerogel shrinkage.

The zein-free starch aerogels [Z0] had higher bulk densities than [Z1] aerogels (*ρ_Bulk_* in Table 1) due to a higher shrinkage of the gels. Nevertheless, the expected decreasing trend of the bulk density with the zein content did not occur with higher zein porogen contents used ([Z2] in Table 1). This behavior might be related to incomplete zein removal (<5% zein remaining). The overall porosities (*ε*) of starch aerogels followed the reverse trend as with the bulk density values and were in the 85–91% range, comparable to those of cancellous human bone [37].

The presence of a mesoporous structure on the obtained aerogel scaffolds was confirmed by N_2_ adsorption-desorption tests. The average pore diameter (d_p_,_BJH_) slightly varied between formulations (16–19 nm), being the highest value for [Z0] aerogels. A decreasing trend was appreciated in the specific pore volume (V_p,BJH_) values as the added zein amount increased (Table 1 and Figure S1). High specific surface areas (A_BET_) were obtained for the manufactured aerogels (143–208 m^2^/g), which correlate with those reported for high amylose content (>40%) starch aerogels [12,33]. The different contributions of pore size ranges to the overall pore volume unveiled the porogen effect in the aerogel morphology. The porogen addition induced an increase in the macropore population (V_p,macro_ in Table 1) of 3.3 and 1.9% for [Z1] and [Z2] formulations, respectively, thus supporting the herein developed method for the production of starch aerogels endowed with dual porosity.

### 2.2. Morphological and Physicochemical Characterization of the Starch Aerogels

The chemical functionality of the starch aerogel formulation [Z0] was similar to that of starch raw material according to the ATR-FT-IR results (Figure S2). Therefore, thermal gelatinization treatment and subsequent supercritical drying unaltered the chemical structure of the corn starch as previously reported for other starch sources [38]. The presence of zein during the aerogel processing did not alter the chemical structure in [Z1] and [Z2] aerogel formulations since no band shifts were detected. Remnants of zein protein in the aerogel structure were detected by the presence of a band at 1540 cm^−1^, corresponding to the amide II stretching mode from the said protein. On the other hand, the native granular structure of raw starch is known to be destroyed during the starch gelatinization and partially recrystallized during the starch retrogradation upon storage [17]. According to the XRD results (Figure S3), the starch aerogel formulations had similar patterns and a reduced crystallinity with respect to the raw starch. 

**Figure 2:**
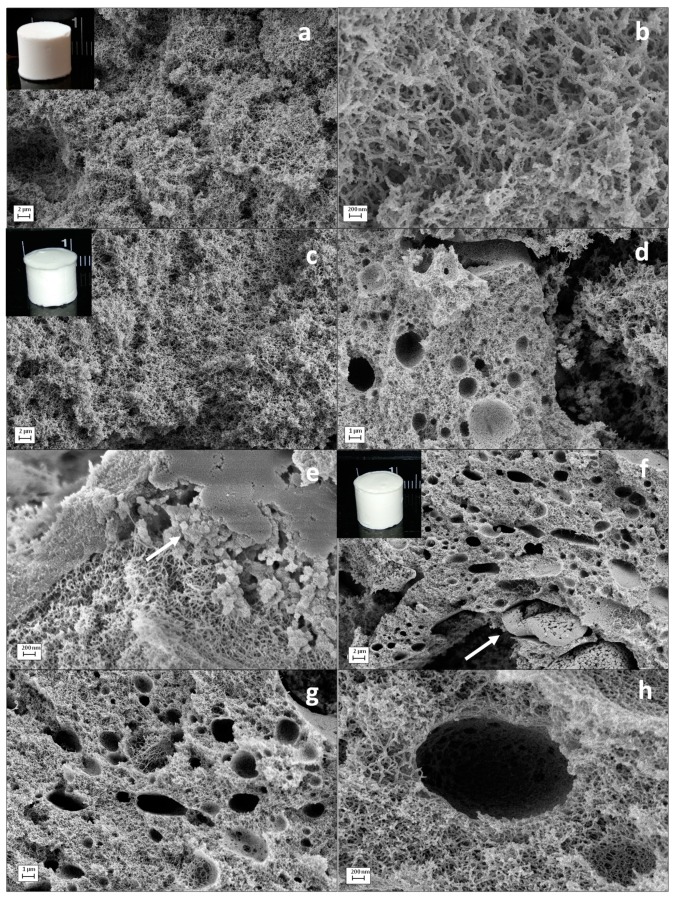
SEM images of cross-sections of the starch aerogels. Characteristic fibrous starch network enlightened by zein-free [Z0] aerogels (**a**,**b**). The porogen addition and subsequent removal caused larger and spherical pores along the inner aerogel architecture for (**c**–**e**) [Z1] and (**f**–**h**) [Z2] aerogel formulations. For these aerogels, residues from zein protein were present, suggesting an incomplete porogen removal (**e**,**f**; white arrow highlighting zein residues). Intakes: Visual appearance of starch aerogel cylinders (**a**) [Z0], (**c**) [Z1], and (**f**) [Z2], respectively.

SEM imaging was performed to evaluate the inner morphology of each aerogel formulation. All starch aerogel formulations were formed by a characteristic fibrous network (Figure 2a–c,f) and typical of high amylose-content starch aerogels [12]. The use of zein porogen during the aerogel processing changed the porous morphology of the material. Starch aerogel processing in the presence of zein led to the formation of pores in the scale of microns built into the characteristic nanoporous backbone of starch aerogels (Figure 2d,f–h). The addition of zein during the aerogel processing resulted in a more open macroporosity and in the presence of spherical macropores with diameters of 1–2 μm into the aerogel structure ([Z1] in Figure 2d,e). This new macropore population was more abundant for the aerogel formulation with higher zein content ([Z2] in Figure 2g). In addition, higher magnifications allowed the identification of granules and smooth surfaces in the macropore walls of [Z2] aerogels corresponding to zein residues and confirming its role in generating this family of pores (Figure 2g,h). The thermal gelation treatment at 121 °C and 1.1 bar increases the water-soluble fraction of zein [39] and uses pHs near the isoelectric point of zein (6-2-6.8) [40,41] so that protein aggregates of ca. 100 nm and thin zein films are formed (Figure 2h). Moreover, the water-insoluble fraction of zein tends to form β-sheet and random coil structures and to promote a higher cross-linking of the zein through disulphide bonds resulting in zein aggregates in the 1–10 µm diameter size [39,40,42] and larger, which are responsible of the large macropores observed in the starch aerogels [Z1] and [Z2] (Figure 2d,g).

**Figure 3:**
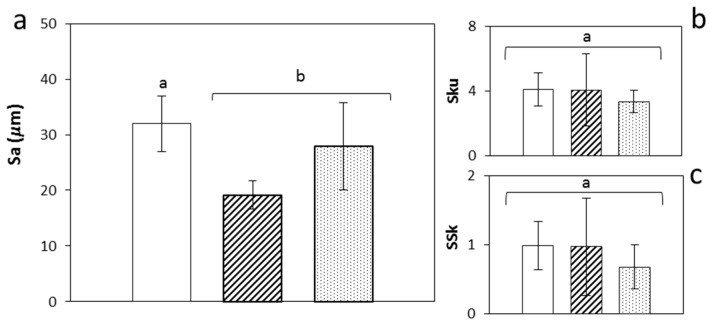
Surface topography of starch aerogels through a contactless 3D-optical profiler. Surface texture parameters: (**a**) arithmetical mean height, (**b**) kurtosis, and (**c**) skewness describing height according ISO 25178. Legend: [Z0] (white bars), [Z1] (black stripes) and [Z2] (black dots). Results were statistically compared (1 way-ANOVA; *p* < 0.05). Equal letter denotes statistically homogeneous groups.

Material surface roughness and topography are amongst the physicochemical properties that conditions the adhesion of cells to the scaffolds among other cell functions [43,44]. A topography for scaffolds combining cells-material contact at the microscale and nanoscale is necessary to reach effective tissue integration [45]. The high porosity of the starch aerogels in the nanoscale (Figure 2) can mimic the extracellular matrix and significantly contribute to the number of anchoring sites in the scaffolds for surface adhesion of cells [43,46], to influence the cell behavior and to promote certain biological pathways for tissue growth [45,47,48,49]. Moreover, the positive effect of surface roughness at the microscale in scaffolds on tissue growth has been clinically confirmed. Height-descriptive 3D parameters were employed for the characterization of the surface topography of aerogels at the macroscale (Figure S4). The surface analysis confirmed the presence of microroughness [43] in every aerogel formulation with Sa values in the 20–30 μm range (Figure 3) with the highest value for [Z0] aerogels and falling in the range promoting the functional attachment of biological tissue [49]. Regardless of the added porogen content, aerogel surface irregularity was defined by a predominance of peaks (Ssk > 0) with a slightly spiked height distribution (Sku > 3) (Figure 3). 

**Figure 4:**
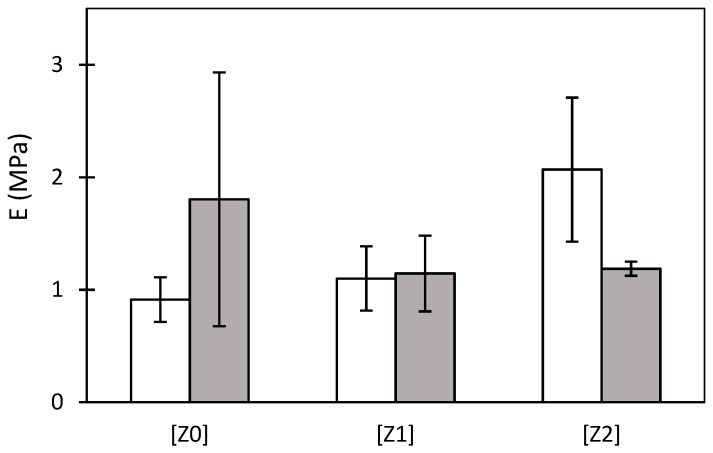
Young’s moduli of the starch aerogels monoliths obtained from different zein porogen contents. Mechanical properties of aerogels were evaluated before (white bars) and after (gray) supercritical sterilization treatment. Results showed no statistical differences (2-way ANOVA), [F_2,10d.f._= 1.37; *p* < 0.05], [F_2,10d.f._= 4.7 × 10^−3^; *p* < 0.05], and [F_2,10d.f._= 3.27; *p* < 0.05] for factors “zein content” and “sterilization treatment”, and its interaction, respectively.

The mechanical compatibility of the starch aerogels with regenerative medicine applications and the effect of the zein porogen on the mechanical properties were studied by compressive mechanical tests. The cylindrical probes experimented plastic deformation (*ca.* 25%) without fracture (Figure S5). The obtained Young’s moduli for all the starch aerogel formulations (E in Figure 4) were in the 0.9–2.1 MPa range, which is a superior mechanical performance compared to other biocompatible polysaccharide-based aerogels [50]. The induction of macropores by zein porogen on the well-defined microstructure of the aerogels did not result in statistical differences (*p* < 0.05) between the formulations regarding their mechanical behavior.

### 2.3. Supercritical CO_2_ Sterilization Treatment Efficacy and Influence on Aerogel Properties

**Figure 5:**
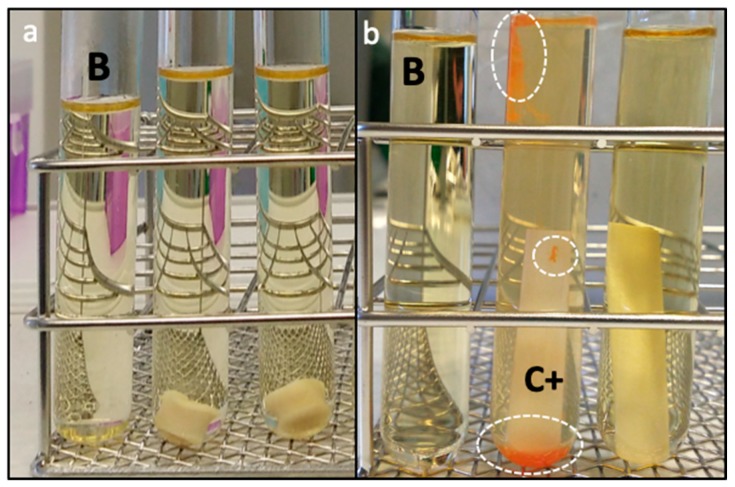
Evaluation of sterilization efficacy of a scCO_2_ treatment on starch aerogels: (**a**) Example of lack of turbidity on TSB tubes containing the aerogel [Z1] after a 7-day incubation period, compared to the blank (B); and (**b**) visual differences between untreated (C+,middle) and scCO_2_ treated *B. atrophaeus* strips (right), compared to the blank (B, left). White circles highlight bacterial growth. The characteristic orange-red color due to the growth of these spores was absent in the scCO_2_ treated strips and in the blank.

The use of aerogels for regenerative medicine applications needs to take into account the sterility requirements for medical devices. After the starch aerogel processing, the aerogels were evaluated regarding their sterility through visual inspections of turbidity of its incubation medium by comparison to the blank (TSB medium). All the tested formulations were sterile using the scCO_2_ method (Figure 5a), regardless their zein content. In this case, sterile conditions were achieved since starch aerogels were gelified under steam sterilization conditions and the solvent exchange to ethanol, being one of the most commonly used antiseptic agent worldwide, promotes the preservation of the sterile conditions. Moreover, during the supercritical drying of the starch gels the high diffusivity of scCO_2_ can penetrate the intricate geometry of nanostructured materials like agglomerates of nanoparticles, cyclodextrins, or aerogels, which have been exploited for certain biomedical applications like drug impregnation, encapsulation, or surface treatments [51,52,53] and was herein exploited to favor the sterilizing effect of scCO_2_. Nevertheless, a specific sterilization method should be developed for bio-based aerogels since sterile conditions during all the aerogel processing steps for other biopolymer sources or the lack of environmental contamination during the handling and/or packaging cannot always be guaranteed.

The scCO_2_ sterilization treatment can overcome the limitations of penetration capacity in porous materials like aerogels, which is encountered in other sterilization techniques like gamma, e-beam and UV-irradiation [30]. Specific scCO_2_ sterilization post-treatment was herein tested for aerogels with hydrogen peroxide (300 ppm) as additive. The turbidity in the tubes containing scCO_2_-treated spore strips was compared with that of untreated strips of the bioindicators and the TSB medium (Figure 5b). No bacterial growth was detected for the scCO_2_-treated strips of *B. atrophaeus* (Figure 5b) and *B. stearothermophilus* (not shown) after the incubation period. However, the developed sterilization method was ineffective against *B. pumilus* strain, the most resistant among the three employed biological indicators, and turbidity was detected in the tube containing the scCO_2_-treated *B. pumilus* strips. An increase in the H_2_O_2_ content would clearly compromise the microbial viability of this microbial strain without major structural modifications on the resulting aerogels [54]. 

The influence of the scCO_2_ sterilization treatment on the starch aerogels was studied regarding morphological, physicochemical and mechanical changes. Low depressurization rates were used after the sterilization treatment to mitigate potential structural damages induced by high venting rates [55]. It is worth mentioning that the supercritical sterilization treatment was challenged against starch aerogels, a particularly sensitive bio-based aerogel, since the textural properties of starch aerogels are influenced by the contact time with scCO_2_ [56].

**Figure 6:**
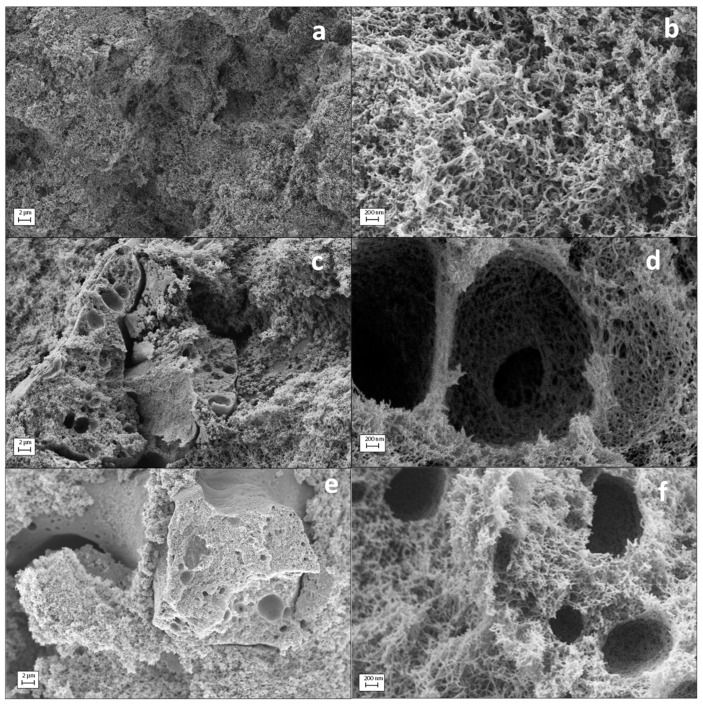
SEM images of cross-sections of sterile starch aerogels. The representative fibrous starch network is maintained after the sterilization treatment (**a**,**b**). The zein effect in (**c**,**d**) [Z1]_S_ and (**e**,**f**) [Z2]_S_ aerogel formulations was homologous to their unsterile aerogel counterparts. Higher magnifications unveiled areas of dual porosity identical to those obtained for untreated aerogels (**d**,**f**).

Starch aerogels maintained their shape and appearance after the sterilization treatment, regardless of the zein content used (Figure 6). However, a noteworthy increase in the shrinkage and a decrease in porosity values were detected after the scCO_2_ sterilization treatment, particularly in [Z0]_S_ (Table 1). The lower gel shrinkage in [Z2]_S_ might be explained by the reinforcement of the pore walls by the zein granules (cf. Section 2.2). The removal of structural water from the starch inner architecture might take place upon processing times over 6 h under a scCO_2_ environment influencing the structure and textural properties of the sterilized aerogel [17]. Moreover, the solubility of water in compressed CO_2_ at the selected sterilization conditions (2.6 g/L [57]) also contributes to this removal of water. Accordingly, a noteworthy decrease in the surface area (A_BET_ in Table 1) and specific pore volume (V_P,BJH_) was observed for [Z0]_s_ starch aerogels. For the starch aerogels processed with zein ([Z1]_S_ and [Z2]_S_), this effect was counteracted by the decrease in the macroporous contribution to the aerogel porosity, as the A_BET_ and V_P,BJH_ values were almost unaltered or even slightly increased (Table 1 and Figure S1). The mechanical behavior of the scCO_2_ treated aerogels was compared to their unsterile counterparts with no statistical differences (*p* < 0.05) obtained between both groups (Figure 4).

### 2.4. Cell Viability Assay

**Figure 7:**
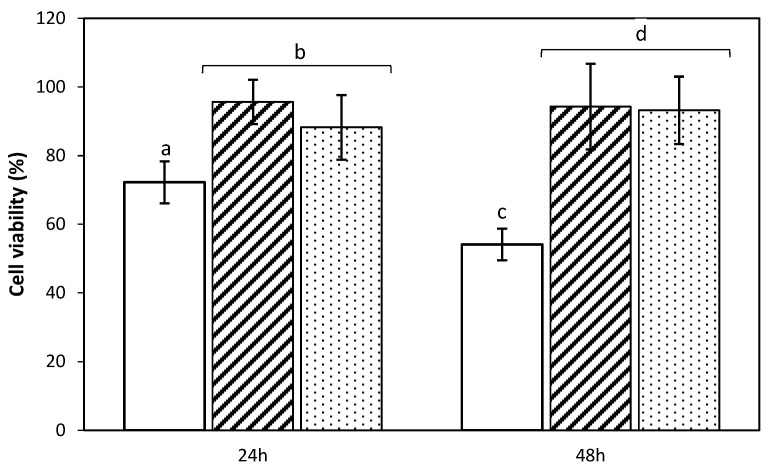
MSCs cell viability studies determined by WST-1 test after 24 and 48 h of contact with sterile starch aerogels formulations: viability was expressed in percentage. Legend: [Z0] (white bars), [Z1] (black stripes), and [Z2] (black dots). Results were statistically compared (1 way-ANOVA; *p* < 0.05). Equal letter denotes statistically homogeneous groups.

Following the scCO_2_ sterilization treatment, the biocompatibility of the aerogel formulations was determined by evaluating the viability of MSCs cells after 24 and 48 h of culture in the presence of the scaffolds. The cell proliferation WST-1 reagent was employed for quantification of cell viability since the enzymatic degradation of the said reagent into formazan directly correlates to the number of metabolically active cells. Cell viabilities close to 100% were obtained for the aerogels formulations modified with zein ([Z1]_S_, [Z2]_S_), regardless of the period of culture tested (Figure 7). Nevertheless, the lowest values were identified for [Z0]_S_ formulation, ranging 50–70% of viability. Cell viability results are suitable and fall in the same range as for other starch-based materials for biomedical applications and for other biomaterials proposed as bone scaffolds [58,59,60]. The statistical analysis unveiled significant differences between formulations, thus supporting the employed processing approach for obtaining dual-porous aerogel scaffolds from a biological point of view. 

## 3. Materials and Methods

### 3.1. Materials

Native corn starch (52.6% amylose content) was provided by Roquette Frères S.A. (Lestrem, France). Zein (m.p. 266–283 °C, size of dry agglomerates by the sieving method: 557 ± 208 µm) was purchased from Sigma-Aldrich, Inc. (Madrid, Spain). CO_2_ (purity of >99.9%) was supplied by Praxair, Inc. (Madrid, Spain). Sterilization reel were purchased from E-line S.r.l. (Torre Pallavicina, Italy). Commercial spore strips with 10^6^ spores of *Bacillus stearothermophilus* (ATCC 7953) and *Bacillus pumilus* (ATCC 27142) were purchased from Sigma-Aldrich, Inc. (Madrid, Spain) and *Bacillus atrophaeus* (cell line 9372) spores were obtained from Crosstex International, Inc. (Rush, NY, USA). Absolute ethanol (EtOH) was provided by VWR (Radnor, PA, USA). *T. Trypticase* soy broth (TSB) medium was purchased from BIOKAR Diagnosis (Pantin, France) and hydrogen peroxide 30% (*v*/*v*) from Sigma-Aldrich, Inc. (Madrid, Spain). Human bone marrow-derived mesenchymal stem cells (ATCC^®^ PCS-500-012™) were obtained from the American Type Culture Collection (ATCC, Manassas, VA, USA). Minimum Essential Medium Alpha (αMEM) and Opti-MEM^TM^ were purchased from Thermo Fisher Scientific (Waltham, MA, USA). Fetal bovine serum and penicillin 10,000 U/mL–streptomycin 10 mg/mL were supplied by Sigma-Aldrich (Saint Louis, MO, USA). Cell proliferation reagent WST-1 was supplied by Roche (Basel, Switzerland).

### 3.2. Starch Aerogels Processing

Corn starch aerogels were produced according to the following procedure. Starch aqueous dispersions of 10% (*w*/*w*) were prepared with zein added as porogen agent in different zein-to-starch weight ratios (0:1, 1:2, and 1:1). The mixtures were autoclaved at 121 °C and 1.1 bar for 20 min (Raypa Steam Sterilizer, Terrassa, Spain) for starch gelatinization. The resulting aqueous solutions were stirred at 1500 rpm for 30 s with a magnetic stirrer (IKA RCT basic, Staufen, Germany) to ensure homogeneity, and then dosed in cylindrical polypropylene molds (L:11 mm, D: 9 mm) and settled for gelation for 20 min at room temperature. Gels were then stored at 4 °C for 48 h to enhance the starch retro-gradation. Afterwards, starch-based gels were immersed in absolute ethanol for solvent exchange and porogen (zein) leaching. To ensure the removal of the zein, the solvent was replaced until no zein was detected in the ethanol by UV-Vis spectrophotometry (8453, Agilent, Santa Clara, CA, USA) at a wavelength of λ = 279 nm and at room temperature. Prior to the measurements, a calibration curve in 70% (*v*/*v*) ethanol was validated (R^2^ > 0.999) in the 0.1–0.7 mg/mL range of zein (Figure S6). Starch alcogels were dried by supercritical drying to obtain the aerogels. Briefly, the gels were loaded in a 100 mL-stainless steel autoclave (Thar Process, Pittsburg, PA, USA) and immersed in 45 mL of absolute ethanol to prevent gel shrinkage and cracks formation [56,61]. Three sequential steps took place under the operating conditions of 40 °C and 130 bar: (1) A continuous CO_2_ flow of 6 g/min for 2.5 h; (2) scCO_2_ environment in the batch mode for 2 h; and (3) a continuous CO_2_ flow of 5 g/min for 1.5 h. Finally, aerogels were obtained after depressurization at a rate of 2 bar/min. Starch aerogels were collected from the autoclave and stored in sealed containers for subsequent characterization. Starch hydrogels with 1:5, 1:3, and 1:0 zein-to-starch weight ratios were also produced following the same gelation method reported above for the sake of visual comparison of physical integrity and homogeneity of the gels.

### 3.3. Supercritical CO_2_ Sterilization Treatment

**Figure 8:**
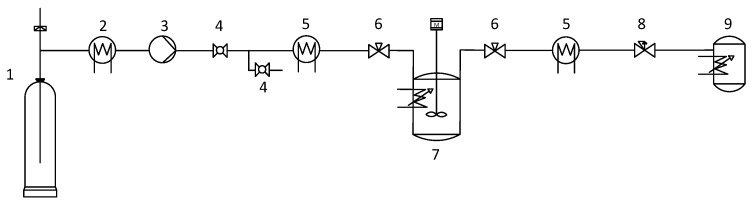
Schematic diagram of the equipment used for the scCO_2_ sterilization treatment. Legend: 1. CO_2_ bottle; 2. chiller; 3. CO_2_ injection pump; 4. ball valves; 5. heat exchangers; 6. heated needle valves; 7. pressure vessel equipped with a stirrer, a refrigeration coil system and a heating jacket; 8. Backpressure regulator; and 9. collector vessel.

The experimental equipment for sterilization is depicted in Figure 8. Briefly, aerogels were placed in triplicate into thermally sealed Tyvek pouches. After placing the aerogels suspended inside a 1.2-L vessel (Parr Instrument Co., Moline, IL, USA), 1 mL of hydrogen peroxide 30% (*v*/*v*) was added at the bottom of the said vessel as an additive to improve the sterilization efficacy specially towards endospores [31,62,63]. Then, the system was heated at 40 °C under a 600 rpm stirring and pressurized until 245 bar at constant CO_2_ flow of 30 mL/min. The setup was maintained under these experimental batch conditions for 6 h. Finally, the system was slowly depressurized at 4 bar/min until reaching atmospheric pressure. 

### 3.4. Physicochemical, Structural and Mechanical Characterization

The skeletal density of the aerogels (*ρ_Skel_*) was determined using a helium-pycnometer (Quantachrome, Boynton Beach, FL, USA) at room temperature (25 °C) and 1.01 bar. Values were obtained from five replicates (standard deviation < 6%). The bulk density of the scaffolds (*ρ_Bulk_*) was determined by measuring the dimensions and weight of the aerogel cylinders. The resulting overall porosity (*ε*) was calculated according to Equation (1).

(1) $$\varepsilon \left(\%\right)=\left(1-\frac{\rho_{Bulk}}{\rho_{Skel}}\right)\times 100$$

The textural properties of the aerogels were determined by N_2_ adsorption-desorption analyses (ASAP 2000 Micromeritics Inc, Norcross, GA, USA). Prior to the measurements, aerogels were outgassed at 80 °C and under vacuum (<1 mPa) for 24 h. The specific surface area (A_BET_) of the aerogels scaffolds were determined by the Brunauer-Emmett-Teller (BET) method. Specific pore volumes (V_p,BJH_) and mean pore diameter (d_p,BJH_) were evaluated using the Barrett-Joyner-Halenda (BJH) method. Based on these results, the contributions (in percentage) of mesopores (2–50 nm range, V_p,meso_) and low macropores (50–100 nm, V_p,lmac_) to the total pore volume were determined. The contribution of macropores population (>50 nm, V_p,macro_) was determined by difference between the total pore volume (V_p_ from Equation (2)) and the mesopore volume (Equation (3)).

(2) $$V_{p}=\left(\frac{1}{\rho_{Bulk}}-\frac{1}{\rho_{Skel}}\right)$$

(3) $$V_{p,\mathrm{meso}}(\%)=\left[\frac{\sum_{Vp}\left(2-50\;nm\right)}{V_{P}}\right]\times 100$$

Infrared Spectroscopy (ATR/FT-IR) was carried out for the chemical characterization of the aerogels using a VARIAN FT-IR 670 spectrometer (Palo Alto, CA, USA) with a Gladi-ATR accessory equipped with a diamond crystal (Pike, Madison, WI, USA). Solid samples of raw materials and aerogels were characterized in the 400–4000 cm^−1^ spectrum range at a resolution of 2 cm^−1^ and using 32 scans. Raw materials and the starch aerogels were also studied by X-ray diffraction (XRD, PW-1710, Philips, Eindhoven, The Netherlands) in the 2–80° 2θ-range, using a 0.05° step, with 3 s of step time and using CuKα1 target radiation.

The structure of the aerogels was evaluated by scanning electron microscopy (SEM, EVO, LS15, Zeiss, Oberkochen, Germany) running at 3 kV. Prior to imaging, aerogels were iridium-sputtered (10 nm thickness). Orography characterization was performed using a contactless 3D-optical profiler (S Neox, Sensofar-Tech, Terrassa, Spain). The arithmetical mean height (Sa) was used to evaluate the surface roughness, being the most common reported parameter. Moreover, the shape of the height distribution of the surface was analyzed thought the skewness (Ssk) and the kurtosis (Sku) in pursuit of differences between aerogel formulations.

A volume reduction of the gels took place during the solvent exchange and the supercritical drying steps with the overall shrinkage volume (ΔV) calculated using Equation (4):

(4) $$\Delta V\left(\%\right)=\left(\frac{V_{0}-V}{V_{0}}\right)\times 100$$

where *V*_0_ denotes the initial volume of the hydrogel and *V* the end volume of the aerogel after processing (subjected either to sterilization treatment or not).

Young’s moduli (*E*) of aerogel cylindrical probes were determined from stress-strain curves under orthogonal compression tests in a tensile bench with a 30 kg load cell (TA.XT*Plus*, Texture Technologies Corp. and Stable Micro Systems, Ltd., Godalming, UK) at a crosshead speed set to 1 mm/min. The compression tool was coated with a Teflon film to prevent the aerogel probes from sticking. Tests were performed at room temperature (26 °C) under atmospheric pressure and 45% relative humidity. Each formulation was evaluated in triplicate.

### 3.5. Microbiological Assessment

The efficacy of the supercritical sterilization method was evaluated using spore strips (10^6^ spores/strip) of three biological indicators typically used in standard procedures for evaluating efficacy in steam sterilization (*B. stearothermophilus* -ATCC 7953-) [64], ethylene oxide or dry heat sterilization (*B. pumilus* -ATCC 27142-) [65] and for radiation sterilization (*B. atrophaeus* -cell line 9372-) [66]. The bacterial growth was evaluated through turbidity tests after incubation (Raypa Digital Incubators, Terrassa, Spain) of each strip in 10 mL of trypticase soy broth (TSB) medium under a sterile environment (flame) and without stirring at two temperatures, 37 and 55 °C, corresponding to the optimal growing temperature for *B. pumilus* and *B. atrophaeus*, and for *B. stearothermophilus*, respectively. On the other hand, every treated aerogel specimen was incubated in 10 mL of TSB medium at 37 °C following the latter sterile seeding protocol. TSB medium (blank), untreated spore strips (positive control) and untreated aerogels were incubated at the same conditions as controls of the process. Microbial growth was visually examined for seven days as defined in the standard protocol and additionally for 15 and 30 days after the scCO_2_ treatment to confirm the obtained results.

### 3.6. Cell Viability Assay

Human bone marrow-derived mesenchymal stem cells (6500 cells/cm^2^, passage 4) were seeded in 24-well plates in αMEM supplemented with 15% fetal bovine serum, penicillin 100 U/mL and streptomycin 100 μg/mL. Cells were incubated for 24 h at 37 °C in a humidified atmosphere with 5% CO_2_. Sterile [Z0]_S_, [Z1]_S_ and [Z2]_S_ scaffolds (9–12 mg) were placed in Transwells in quadruplicate with 200 µL of Opti-MEM^TM^. The Transwells were introduced in the wells with cells containing 800 µL of Opti-MEM^TM^. Positive controls of cells with 1000 µL of Opti-MEM^TM^ and blanks of 1000 µL of Opti-MEM^TM^ both in quadruplicate were included and treated in the same way. After 24 h of culture, the Transwells were removed, supernatants were taken and kept at −80 °C leaving 250 μL in the wells. 25 μL of WST-1 reagent were added to the wells to measure the cell viability. The plate was incubated for 2 h at 37 °C in a humidified atmosphere with 5% CO_2_ and then shaken thoroughly for 1 min. 110 µL were transferred in duplicates to a 96-well plate and the absorbance was measured at the wavelength of 450 nm in a microplate reader (Infinite^®^ M200, Tecan Group Ltd., Männedorf, Switzerland).

### 3.7. Statistical Analysis

All results were expressed as mean ± standard deviation. Statistical analyses of surface roughness values (1-way ANOVA), cell viability (1-way ANOVA), and mechanical behavior (2-way ANOVA) were performed using Statistica v8.0 software (Stat Soft Inc., Tulsa, OK, USA) followed by the post hoc Tukey HSD multiple comparison test.

## 4. Conclusions

Sterile corn starch-based aerogels with tailor-made porosities were obtained by an innovative multi-step integrating approach. Macroporosity of 1–2 µm in starch aerogels was induced by the addition of zein, a biocompatible porogen, without involving an extra leaching step, like in other conventional practices. The design and manufacturing of the aerogels using this technological platform has clear advantages, as compared to the hitherto tested methods for the induction of well-integrated macropores in the inner mesoporous backbone of aerogels. The optimized process for scCO_2_ treatment of aerogels ensured an effective sterilization of the end nanostructured materials. Textural properties of the aerogels changed after the sterilization treatment, although the mechanical performance of the treated material was not compromised. The biocompatibility of the sterile aerogel scaffolds showed promising values (>80% cell viability) for the aerogels endowed with dual porosity. The remarkable potential of this supercritical CO_2_ technology is not restricted to sterilization ability purposes, but can also be exploited for the preservation of the physicochemical properties of the biomaterial to be treated, including the complex nanoarchitectures of aerogels. Overall, the herein presented processing method is compatible with the production of bio-based aerogels with dual porosity for regenerative medicine purposes. For the sake of economy of the process, the integration of supercritical CO_2_-based drying and sterilization methods is of interest for further studies.

## Figures and Tables

**Table 1:** Morphological and textural properties of starch-based aerogels processed using different zein porogen contents. Notation: [Z0] = Starch aerogels; [Z1] = Starch aerogels gelified in a 1:2 zein-to-starch weight ratio; and [Z2] = Starch aerogels gelified in a 1:1 zein-to-starch weight ratio. Subscript “S” denotes sterile aerogels obtained by scCO_2_ treatment.

Aerogel	*ρ_Bulk_* (g/cm^3^)	*ρ_Skel_* (g/cm^3^)	*ε* (%)	ΔV (%)	A_BET_ (m^2^/g)	V_p,BJH_ (cm^3^/g)	d_p,BJH_ (nm)	V_p_ (cm^3^/g)	V_p,meso_ (%)	V_p,lmac_ (%)	V_p,macro_ (%)
[Z0]	0.166 ± 0.003	1.490 ± 0.059	88.9 ± 0.2	39.1 ± 2.7	183 ± 9	1.01 ± 0.05	19.1 ± 1.0	5.35	14.2	4.7	85.8
[Z0]_S_	0.304 ± 0.009	1.541 ± 0.045	80.3 ± 0.6	65.8 ± 2.2	130 ± 6	0.84 ± 0.04	22.9 ± 1.2	2.64	20.8	10.9	79.2
[Z1]	0.135 ± 0.005	1.498 ± 0.059	91.0 ± 0.3	25.2 ± 5.0	208 ± 10	0.98 ± 0.05	16.5 ± 0.8	6.74	10.9	3.5	89.1
[Z1]_S_	0.203 ± 0.016	1.512 ± 0.038	86.6 ± 1.1	46.6 ± 7.9	264 ± 13	1.25 ± 0.06	16.1 ± 0.8	4.26	23.6	4.9	76.4
[Z2]	0.189 ± 0.007	1.423 ± 0.055	86.7 ± 0.5	21.7 ± 4.3	143 ± 7	0.74 ± 0.04	16.9 ± 0.8	4.59	12.3	3.6	87.7
[Z2]_S_	0.214 ± 0.016	1.431 ± 0.065	85.1 ± 1.0	25.2 ± 3.5	146 ± 7	1.00 ± 0.05	21.7 ± 1.1	3.97	16.7	8.2	83.3

## Supplementary Materials

The following figures are available online, Figure S1: BJH-pore size distributions from the N_2_-desorption curves of the (a) unsterile and (b) sterile aerogel formulations. Figure S2: ATR-IR spectra of raw materials (zein and starch) and different starch aerogel formulations ([Z0], [Z1] and [Z2]). Figure S3: XRD diffraction patterns for raw materials {(a) starch and (b) zein} and starch aerogel formulations {(c) [Z0], (d) [Z1] and (e) [Z2]}. Figure S4: Surface 2D mapping of the three starch aerogels formulations ([Z0], [Z1], [Z2]) obtained from a contactless 3D-optical profiler. Figure S5: Stress-strain curves of the obtained aerogels under orthogonal compression tests with a 30 kg load cell at a crosshead speed of 1 mm/min. Figure S6: Calibration curve of zein in 70% (*v*/*v*) ethanol in the 0.1–0.7 mg/mL range (R^2^ > 0.999). This curve was used for zein detection during the simultaneous solvent exchange-porogen leaching step during starch aerogel processing.
