# Supplementary material for: Sterile and Dual-Porous Aerogels Scaffolds Obtained through a Multistep Supercritical CO2-Based Approach
Source: Molecules. 2019 Mar 1;24(5):871. doi: 10.3390/molecules24050871 (PMC6429194; doi:10.3390/molecules24050871)
Supplement: Supplementary file 1 [file molecules-24-00871-s001.pdf]

Supplementary Information

Sterile and dual-porous aerogels scaffolds obtained through a multistep supercritical CO_2-based approach

Víctor Santos-Rosales[1], Inés Ardao[2], Carmen Alvarez-Lorenzo[1], Nilza Ribeiro[3], Ana L. Oliveira[3] and Carlos A. García-González[1*]

1. Departamento de Farmacología, Farmacia y Tecnología Farmacéutica, R+D Pharma group (GI-1645), Facultad de Farmacia and Health Research Institute of Santiago de Compostela (IDIS), Universidade de Santiago de Compostela, E-15782 Santiago de Compostela, Spain.
2. BioFarma Research group, Centro Singular de Investigación en Medicina Molecular y Enfermedades Crónicas (CiMUS), Universidade de Santiago de Compostela, E-15782, Santiago de Compostela, Spain
3. CBQF - Centro de Biotecnologia e Química Fina - Laboratório Associado, Escola Superior de Biotecnologia, Universidade Católica Portuguesa, 4200-375 Porto, Portugal.

* Correspondence: carlos.garcia@usc.es; Tel.: +34-881-814882

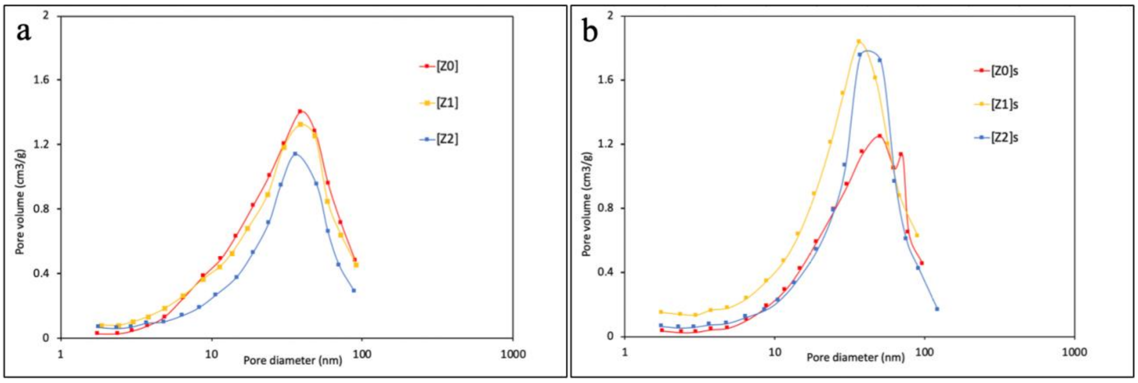

Figure S1: BJH-pore size distributions from the N_2-desorption curves of the (a) unsterile and (b) sterile aerogel formulations.

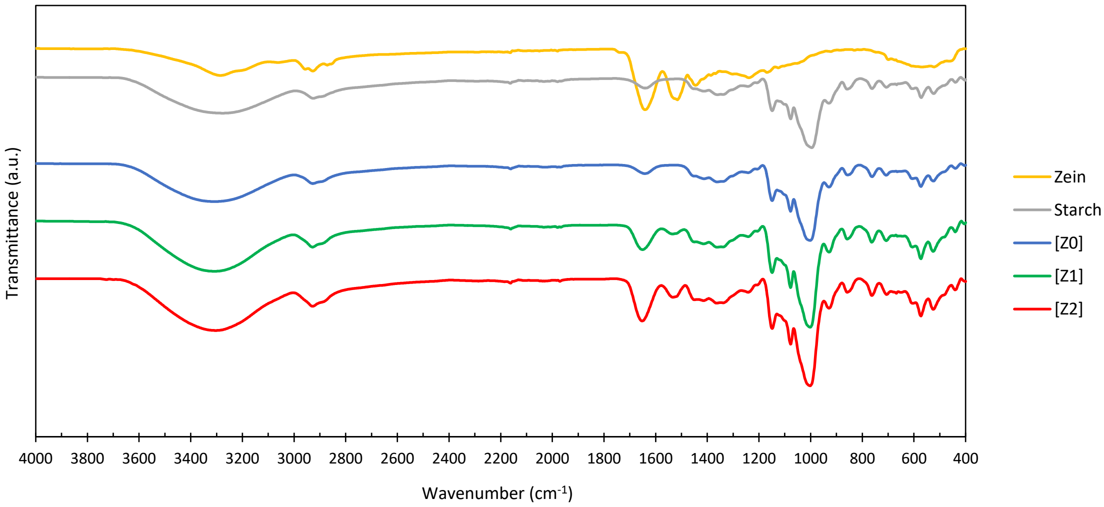

Figure S2: ATR-IR spectra of raw materials (zein and starch) and different starch aerogel formulations ([Z0], [Z1] and [Z2]).

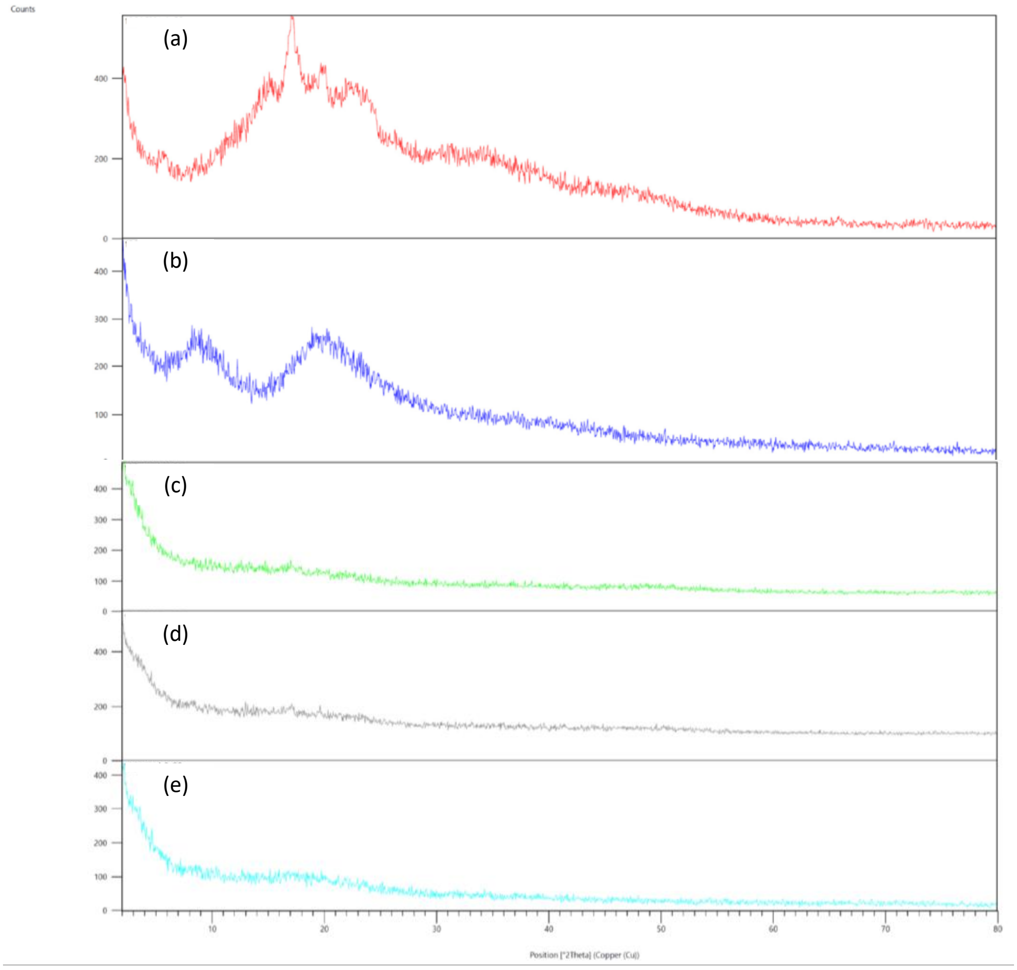

Figure S3: XRD diffraction patterns for raw materials {(a) starch and (b) zein} and starch aerogel formulations {(c) [Z0], (d) [Z1] and (e) [Z2]}.

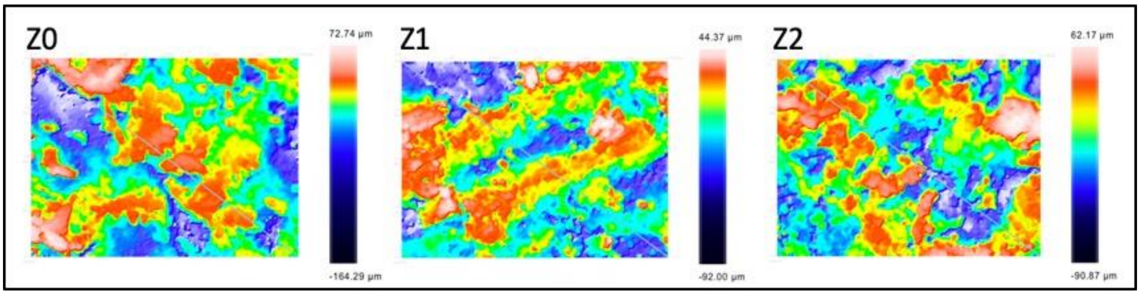

Figure S4: Surface 2D mapping of the three starch aerogels formulations ([Z0], [Z1], [Z2]) obtained from a contactless 3D-optical profiler.

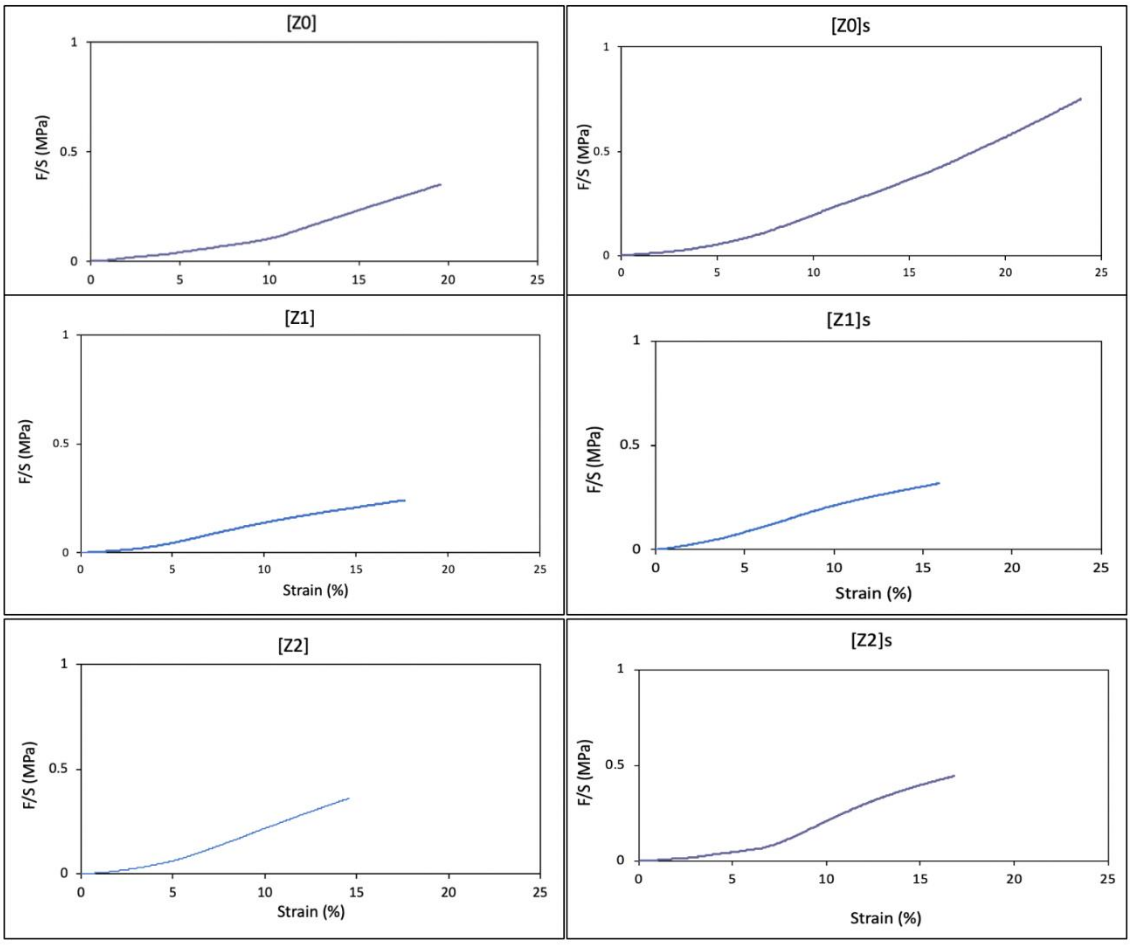

Figure S5: Stress-strain curves of the obtained aerogels under orthogonal compression tests with a 30 kg load cell at a crosshead speed of 1 mm/min.

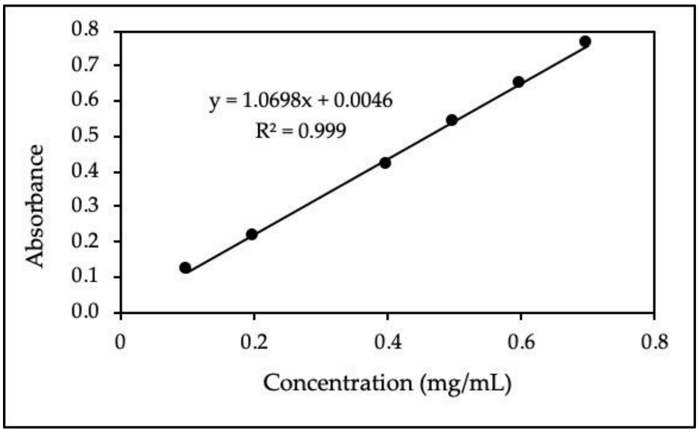

Figure S6: Calibration curve of zein in 70% (v/v) ethanol in the 0.1-0.7 mg/mL range (R^2> 0.999). This curve was used for zein detection during the simultaneous solvent exchange-porogen leaching step during starch aerogel processing.
